# Verrucous Carcinoma of the Lower Lip: A Rare Case Mimicking Benign Lesion

**DOI:** 10.3390/jcm14248763

**Published:** 2025-12-11

**Authors:** Dong Gyu Kim, Kyung Ah Lee

**Affiliations:** Department of Plastic and Reconstructive Surgery, Inje University Haeundae Paik Hospital, Busan 48108, Republic of Korea; h80837@paik.ac.kr

**Keywords:** verrucous carcinoma, advancement flap, histopathologic diagnosis

## Abstract

**Background:** Verrucous carcinoma (VC) is a rare, well-differentiated subtype of squamous cell carcinoma characterized by slow growth and local invasiveness. Although it can arise in various anatomical regions, involvement of the lip is uncommon. Because VC may clinically resemble benign verrucous lesions such as squamous cell papilloma, accurate diagnosis is often delayed. This case report aims to illustrate the diagnostic pitfalls encountered when lower-lip VC is managed as a benign verrucous lesion and to emphasize the need for adequately deep or excisional biopsy in persistent lesions that fail to respond to conservative treatment. **Methods:** We report the case of a 75-year-old man who presented with a persistent, cauliflower-like lesion on the lower lip initially diagnosed as verruca. Despite repeated cryotherapy, the lesion enlarged. Wide local excision was performed under general anesthesia, and frozen biopsy suggested malignancy. The resultant defect was reconstructed using a step-ladder advancement flap designed to preserve lip symmetry and function. **Results:** Histopathologic examination revealed a well-differentiated squamous epithelium with parakeratinized invaginations extending into the stroma, confirming VC. The postoperative course was uneventful, with preserved oral competence and no evidence of recurrence during follow-up. **Conclusions:** Verrucous carcinoma of the lip can be misdiagnosed as a benign papillomatous or verrucous lesion, particularly when only a superficial biopsy is obtained and management relies on prolonged conservative therapy such as repeated cryotherapy. Persistent verrucous lesions of the lip that do not respond to an apparently adequate course of treatment should prompt reconsideration of the diagnosis and performance of an adequately deep or excisional biopsy. Complete excision with negative margins remains the treatment of choice, and increased clinical awareness, together with careful histopathologic evaluation, are essential for early detection and appropriate management, ultimately improving patient outcomes and minimizing morbidity.

## 1. Introduction

Verrucous carcinoma is a rare, well-differentiated squamous cell carcinoma (SCC) manifested as an exophytic or papillary projection usually on a mucosal surface [1].

It is characterized by slow growth, local invasiveness, minimal cytologic atypia, and an extremely low rate of metastasis [2,3]. These clinicopathologic features have been confirmed in larger series and recent reviews on oral verrucous carcinoma [4,5]. Histopathologically, verrucous carcinoma is characterized by well-differentiated squamous epithelium forming broad, bulbous rete ridges with a smooth, broad pushing rather than irregular infiltrative interface with the underlying stroma, together with a thick parakeratotic surface and papillomatosis. These architectural and cytologic hallmarks help distinguish VC from benign verruciform lesions such as verruca vulgaris, squamous papilloma, and verrucous or atypical verrucous hyperplasia, as well as from invasive well-differentiated squamous cell carcinoma, which typically shows more irregular infiltrative nests and greater nuclear atypia. Extraorally, it can occur in any part of the body, a common site being the anogenital region [6]. Intraorally, the buccal mucosa and gingiva are the common site. In contrast, involvement of the lip is exceptionally rare and has been reported only sporadically in small case series and isolated case reports, representing a very small fraction of oral verrucous carcinoma cases [7,8,9,10,11].

Verruca vulgaris is a common viral wart of the skin caused by human papillomavirus infection and typically presents as a hyperkeratotic papule with acanthosis, a prominent granular layer, and koilocytosis. In contrast, oral squamous papilloma is a benign exophytic mucosal lesion composed of papillary fronds with fibrovascular cores lined by stratified squamous epithelium. Although both can present clinically as verruciform lesions, they are distinct entities with different usual locations, etiologies, and histologic features.

Although true malignant transformation of a benign verruca into VC appears to be very uncommon and is not well documented, several reports have highlighted that lesions initially interpreted as verruca or other benign verruciform lesions may later be reclassified as VC once a deeper or more representative biopsy is obtained. Persistent verrucous lesions of the lip that do not respond to an adequate course of conservative therapy should therefore raise suspicion for an underlying malignancy, including verrucous carcinoma. In such situations, reliance on superficial or partial biopsy alone may be misleading, because the diagnostic broad pushing invasion front can be missed and the specimen misclassified as benign hyperkeratosis or papilloma. Referral for surgical evaluation and histopathologic assessment of a sufficiently deep or excisional specimen is therefore recommended rather than indefinite continuation of conservative measures.

Here, we present a rare case of verrucous carcinoma involving the lower lip that was initially diagnosed as verruca on superficial punch biopsy and treated with repeated cryotherapy before definitive excision. The aim of this case report is to illustrate the diagnostic pitfalls of managing a persistent verrucous lip lesion as a benign wart on the basis of superficial biopsy findings and to highlight the importance of timely deep or excisional biopsy, followed by complete excision with appropriate reconstruction, when a presumed benign lesion fails to respond to treatment. In addition, this case is noteworthy for the prolonged period of misdiagnosis and conservative management based on superficial biopsies, the clear clinicopathologic discrepancy between the initial benign diagnoses and the final excision specimen, and the favorable functional outcome after step-ladder advancement flap reconstruction of a relatively large central lower-lip defect with 18 months of recurrence-free follow-up.

## 2. Case Description

**Figure 1:**
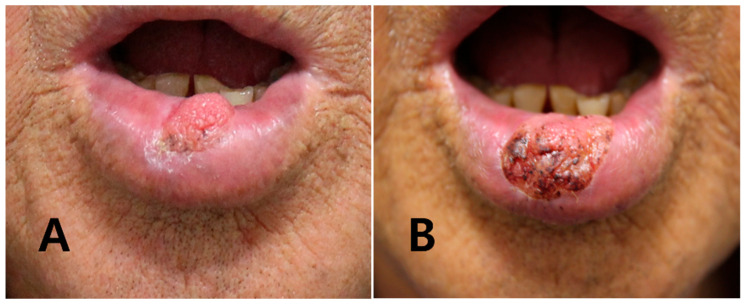
Chronologic clinical photographs at the dermatology clinic. (**A**) Initial presentation showing a small verrucous papule on the central lower lip. (**B**) After approximately one year of repeated liquid nitrogen cryotherapy, the lesion has evolved into a larger cauliflower-like verrucous mass without complete resolution.

A 75-year-old man was referred to our department for evaluation of a persistent verrucous lesion on the lower lip. The lesion had first appeared as a small papule on the central lower lip and had slowly enlarged over several months, with occasional crusting but no significant pain or bleeding (Figure 1A). At the referring dermatology clinic, an initial punch biopsy that sampled only the superficial to mid dermis showed verrucous acanthosis with hyperkeratosis and papillomatosis, without broad pushing rete ridges, deep epithelial downgrowths, or significant cytologic atypia, and was interpreted as verruca. Because the lesion was initially small, asymptomatic, and reported histologically as a benign verrucous lesion, the dermatologist elected to treat it as a viral wart with cryotherapy. The patient subsequently underwent a total of 12 liquid nitrogen cryotherapy sessions at intervals of approximately 2–4 weeks over a year, but the lesion failed to resolve and gradually increased in size, becoming a larger cauliflower-like verrucous mass (Figure 1B). A repeat punch biopsy at the same clinic yielded similar superficial verruciform changes and was again reported as a benign verrucous lesion. Despite this, the lesion continued to enlarge and became more cauliflower-like in appearance. The patient had a history of former smoking for 30 years and prediabetes. Human papillomavirus testing was not performed. Because the lesion remained persistent and progressively enlarged despite an apparently adequate course of conservative therapy, the patient was referred for surgical evaluation after 20 months of dermatologic treatment.

**Figure 2:**
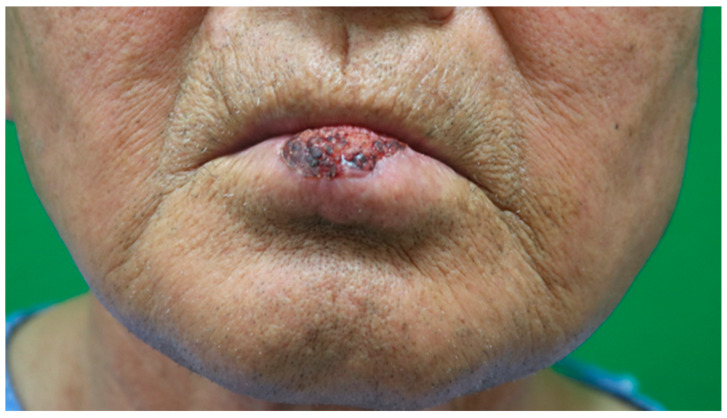
Presentation at the plastic surgery clinic. Cauliflower-shaped, exophytic verrucous lesion measuring approximately 2.5 cm × 1.7 cm on the central lower lip, with a whitish papillomatous surface and relative preservation of the surrounding mucosa.

**Figure 3:**
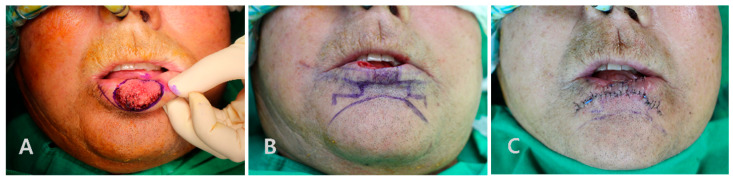
Intraoperative views of wide excision and step-ladder advancement flap reconstruction. (**A**) Preoperative marking of the verrucous lesion on the central lower lip in the operating room. (**B**) Operative field after wide local excision with an approximately 5 mm clinical margin, showing the resulting full-thickness central defect and the design of the step-ladder advancement flap along the lower labial skin on both sides of the defect. (**C**) Immediate postoperative appearance after medial advancement of the step-ladder flaps and layered closure, demonstrating restoration of the lower-lip contour without distortion of the oral commissures.

On presentation to our department, physical examination revealed a cauliflower-shaped, exophytic lesion measuring approximately 2.5 cm × 1.7 cm in the central lower lip, with a whitish papillomatous surface and relative preservation of the surrounding mucosa (Figure 2). No cervical lymphadenopathy was palpable. Under general anesthesia with nasotracheal intubation, wide local excision of the lesion was performed with a clinical margin of approximately 5 mm, including full-thickness resection of the involved vermilion and underlying orbicularis oris muscle (Figure 3A). Intraoperative frozen-section analysis of the central lesion showed a verruciform squamous proliferation with marked acanthosis, elongation and broadening of rete ridges, and focal architectural irregularity suggestive of squamous cell carcinoma, although a definitive distinction between VC and well-differentiated SCC was deferred to permanent paraffin sections. Frozen sections of the circumferential and deep margins were free of tumor. The final defect measured approximately 3.0 × 2.0 cm and involved a substantial portion of the central lower lip, corresponding to about 60% of the total lower-lip length.

Given the size and central location of the defect, we elected to reconstruct it using a step-ladder advancement flap. Two rectangular advancement limbs were designed along the lower labial skin on each side of the defect in a step-ladder configuration, oriented obliquely at approximately 45 degrees to the defect margins and aligned with relaxed skin tension lines (Figure 3B). After careful subcutaneous dissection, taking care to preserve the inferior labial artery and to avoid violating the labiomental crease, the flaps were advanced medially in a stepwise fashion and inset to recreate the lower-lip contour and oral sphincter. Layered closure was performed with vicryl 5-0 and 6-0 absorbable sutures for the muscle and mucosa and silk 6-0 for the skin and vermilion (Figure 3C). The oral commissures were not distorted, and lip length and symmetry were adequately maintained.

We maintained a full liquid diet for 2 days and applied ointment without dressing due to the lesion’s location. As no other complications such as bleeding or hematoma occurred, the drain was removed. A soft diet was initiated after 3 days. All stitches were re-moved, and the patient was discharged after 7 days.

## 3. Results

**Figure 4:**
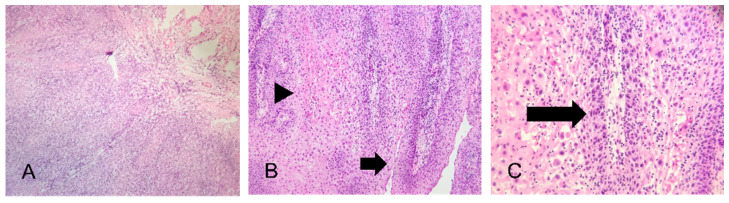
Histopathologic features of the excised verrucous carcinoma. (**A**) Low-power view showing an exo–endophytic verruciform squamous proliferation with broad, bulbous rete ridges forming a pushing interface with the underlying stroma (H&E, ×40). (**B**) Medium-power view demonstrating verruciform projections covered by thick parakeratotic surface keratin (arrows). Deep cleft-like parakeratotic invaginations extend into the underlying fibrous stroma and are bordered by broad bulbous rete ridges (arrowheads) (H&E, ×100). (**C**) High-power view of the epithelial downgrowths showing well-differentiated squamous cells with relatively uniform, bland nuclei and minimal cytologic atypia (arrows), with only rare mitotic figures and no foci of conventional infiltrative squamous cell carcinoma, consistent with pure verrucous carcinoma (H&E, ×200).

Histopathologic examination of the excised specimen revealed a well-differentiated verruciform squamous proliferation with an exo–endophytic architecture (Figure 4A–C). Broad, bulbous rete ridges extended deeply into the underlying stroma and formed a pushing rather than infiltrative interface at the tumor–stromal junction. The surface epithelium showed marked parakeratosis and papillomatosis, with deep cleft-like parakeratotic invaginations extending into a fibrous, chronically inflamed stroma. Cytologic atypia was minimal, and mitotic figures were rare. No irregular jagged infiltrative nests or destructive stromal invasion typical of conventional well-differentiated squamous cell carcinoma were identified, and the tumor–stromal interface remained broad and pushing throughout. No foci of conventional infiltrative squamous cell carcinoma were identified, thereby excluding hybrid verrucous carcinoma. These features were consistent with verrucous carcinoma of the lower lip. On histologic grounds, the main differential diagnoses included verruca vulgaris, squamous papilloma, verrucous and atypical verrucous hyperplasia, pseudocarcinomatous (pseudoepitheliomatous) hyperplasia, and well-differentiated squamous cell carcinoma.

**Figure 5:**
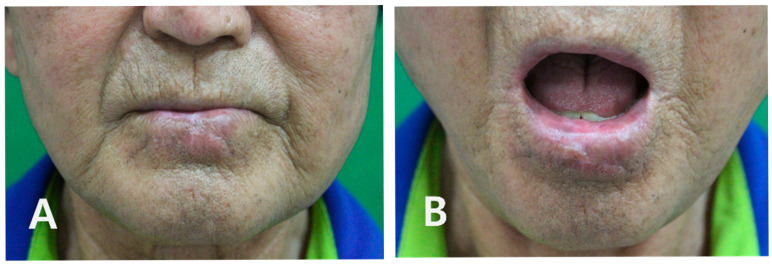
Long-term functional and aesthetic outcome at 18-month follow-up. (**A**) Frontal view with the mouth closed, demonstrating a well-healed linear scar, preserved lower-lip contour, and no visible distortion of the oral commissures. (**B**) Frontal view with the mouth open, showing adequate oral competence without drooling, normal speech articulation, and no evidence of recurrent verrucous lesions.

The postoperative course was uneventful. The flap remained entirely viable, and there were no wound complications such as hematoma, dehiscence, or infection. A full liquid diet was maintained for 2 days, followed by a soft diet from postoperative day 3, and sutures were removed on postoperative day 7. The patient was followed clinically every 3 months during the first year and every 6 months thereafter. At each visit, the lower lip and oral cavity were inspected, and the cervical lymph nodes were palpated; no suspicious nodal enlargement was detected. Additional imaging (contrast-enhanced CT) was performed at 3 months and showed no evidence of metastatic disease. There was no subsequent clinical suspicion of recurrence, so further imaging was not obtained. At the latest follow-up, 18 months after surgery, there was no evidence of local recurrence or regional metastasis, and the patient reported satisfactory oral competence without drooling, normal speech articulation, and acceptance of the cosmetic outcome (Figure 5A,B).

## 4. Discussion

Verrucous carcinoma represents a distinct clinicopathologic subtype of squamous cell carcinoma that usually carries a favorable prognosis when adequately excised. Despite its histologic malignancy, it behaves in a predominantly locally invasive manner without distant spread [2,3]. Within the oral cavity, verrucous carcinoma accounts for approximately 2–12% of all SCCs according to population-based and institutional series [2,5,6]. Head and neck registries and additional epidemiologic studies have confirmed that VC remains a small subset of all SCCs [12,13]. Large oral VC cohorts also emphasize its overall rarity and mention lip involvement only sporadically [5].

Case reports have documented verrucous carcinoma of the lower lip as an uncommon presentation in individual patients [7,8]. Similar lower-lip lesions have been described by other groups, further underscoring that this site is rarely affected [9,10,11].

Clinically, lip verrucous carcinoma often presents as a slowly enlarging, exophytic, white or gray mass with a rough or papillomatous surface. Because of its indolent nature, diagnosis is often delayed, and the lesion may be mistaken for benign conditions such as verruca vulgaris, pseudoepitheliomatous hyperplasia, or chronic traumatic ulcer. The differential diagnosis should also include conventional SCC and keratoacanthoma, which exhibit more aggressive cytologic atypia and destructive invasion [13].

From a histopathologic standpoint, VC must be distinguished from several verruciform or keratotic lesions, including verruca vulgaris, squamous papilloma, verrucous and atypical verrucous hyperplasia, pseudocarcinomatous (pseudoepitheliomatous) hyperplasia, and well-differentiated squamous cell carcinoma. Verruca vulgaris typically shows a prominent granular cell layer and koilocytosis without broad pushing rete ridges, whereas squamous papilloma exhibits exophytic papillary fronds with fibrovascular cores rather than an exo–endophytic architecture. Verrucous and atypical verrucous hyperplasia share verruciform features but lack the deeply pushing bulbous rete ridges of VC and may not form a continuous broad pushing front. Pseudocarcinomatous hyperplasia often arises in association with chronic inflammation or infection and demonstrates a reactive epithelial proliferation without the uniform pushing border characteristic of VC. In contrast, well-differentiated SCC is characterized by more irregular infiltrative nests, greater cytologic atypia, and higher mitotic activity.

Histopathologically, verrucous carcinoma is characterized by well-differentiated squamous epithelium forming broad, bulbous rete ridges with a pushing rather than infiltrative margin [4,14]. These architectural and cytologic features have been consistently reported in larger series of oral VC [5]. The tumor typically exhibits marked parakeratosis, papillomatosis, and elongated epithelial downgrowths, with minimal mitotic activity and nuclear atypia [4,14]. The absence of dysplastic changes and lack of metastatic lymphadenopathy further supported the typical histologic pattern of verrucous carcinoma.

Hybrid verrucous carcinoma, in which areas of conventional infiltrative squamous cell carcinoma coexist with verrucous carcinoma in the same lesion, has been associated with a higher risk of nodal metastasis and poorer prognosis than pure verrucous carcinoma [15,16]. In our case, no foci of conventional infiltrative squamous cell carcinoma were identified in the excision specimen, thereby excluding a hybrid verrucous carcinoma.

In the present case, the diagnosis of VC was made on morphologic grounds, and immunohistochemical studies were not deemed essential at the time of diagnosis. However, previous reports have suggested that immunohistochemistry may be helpful in challenging cases. VC typically shows a low-to-moderate Ki-67 proliferation index largely confined to the basal and parabasal layers and a p53 expression pattern that is less diffuse than that seen in conventional SCC, which often displays a higher and more widespread proliferative and p53 staining pattern. In addition, p16 and HPV-related markers may assist in distinguishing HPV-driven verruciform lesions from non–HPV-related VC in selected cases. Such ancillary studies can support the distinction between VC, verruca, pseudocarcinomatous hyperplasia, verrucous or atypical verrucous hyperplasia, and well-differentiated SCC when morphology alone is equivocal.

Superficial or inadequate biopsies may show only hyperkeratotic or papillomatous mucosa, resulting in misinterpretation as a benign lesion [2,5]. Therefore, when clinical suspicion persists despite benign histology, complete excision or repeat deep biopsy is recommended to obtain a more representative specimen [2,15]. In the present case, initial incisional biopsies suggested verruca, but the final excision specimen revealed characteristic features of verrucous carcinoma, emphasizing the importance of obtaining an adequately deep and representative sample.

The present case is notable in several respects and adds some points that complement previously reported cases of verrucous carcinoma of the lip. First, the lesion was managed as a benign verrucous lesion for an extended period on the basis of superficial biopsies interpreted as verruca, during which the patient received multiple sessions of cryotherapy despite persistence and gradual enlargement of the lesion. This prolonged misdiagnosis and conservative management highlight the risk of relying solely on partial sampling and clinical appearance in verruciform lip lesions. Second, there was a clear clinicopathologic discrepancy between the initial punch biopsies, which sampled only the exophytic portion of the lesion, and the definitive excision specimen, which demonstrated the classic exo–endophytic architecture and broad pushing front characteristic of verrucous carcinoma. Third, the defect involved a substantial portion of the central lower lip, yet step-ladder advancement flap reconstruction provided a single-stage, local solution with preservation of oral competence, symmetric lip contour, and no evidence of recurrence at 18 months of follow-up.

Most published reports have described lower-lip VC that was recognized as malignant or premalignant shortly after presentation or after a single biopsy, followed by prompt wide excision and reconstruction using a variety of local flap techniques [7,11]. In contrast, our patient’s lesion underwent prolonged conservative management based on superficial biopsies interpreted as benign before a subsequent biopsy raised suspicion of malignancy and definitive excision revealed classical VC. Taken together, these features distinguish the present case from most previously reported lip VC cases and provide additional clinical, surgical, and prognostic insight into the management of persistent verrucous lesions of the lower lip.

Complete surgical excision with histologically negative margins remains the treatment of choice for verrucous carcinoma of the lip, and this approach was sufficient in our patient. Recurrence is usually associated with incomplete removal rather than inherent tumor aggressiveness [4]. Elective neck dissection is generally not indicated because of the extremely low metastatic potential, and careful clinical surveillance of the cervical lymph nodes is usually adequate. Radiotherapy has been used as an alternative or adjunctive modality in selected patients with oral verrucous carcinoma; however, several reports have described anaplastic transformation to poorly differentiated squamous cell carcinoma after irradiation [17]. Accordingly, radiotherapy should be reserved for patients who are not suitable candidates for surgery or have unresectable disease, and its potential risks should be weighed in light of the existing literature rather than the present case.

Reconstruction of lip defects requires careful balance between oncologic safety and restoration of function and appearance. Depending on defect size and location, various techniques have been described, including primary closure and local advancement or rotation flaps, as well as Karapandzic and Abbe–Estlander flaps [18]. Bernard–Webster flaps and staircase or step-ladder advancement flaps have also been used for larger lower-lip defects [8,19].

In our case, the defect involved the central lower lip and accounted for roughly half of the lip length while sparing the commissures. A step-ladder advancement flap was chosen because it allows tension to be distributed across multiple small advancement steps, preserves the position of the oral commissures, and maintains adequate lip length without the need for cross-lip tissue transfer. Compared with alternatives such as Abbe–Estlander or large rotation flaps, this technique provided a single-stage local reconstruction with minimal distortion of the vermilion border and satisfactory restoration of oral competence [8,19].

Long-term follow-up is necessary, as local recurrence can occur months or even years after treatment. In our patient, a structured follow-up schedule with clinical examinations every 3 months during the first year and every 6 months thereafter allowed close surveillance for local recurrence or nodal disease, although none was detected during 18 months of follow-up. Although metastasis is rare, periodic examination of regional lymph nodes is advisable. Future research should focus on clarifying the molecular pathogenesis of verrucous carcinoma, particularly the potential role of HPV infection and chronic mucosal trauma.

## 5. Conclusions

This case illustrates that verrucous carcinoma of the lower lip can be misdiagnosed and managed for an extended period as a benign verrucous lesion when only superficial biopsies are obtained and treatment relies on repeated cryotherapy. Persistent verrucous lesions of the lip that do not resolve despite an apparently adequate course of conservative therapy should prompt reconsideration of the initial diagnosis and raise suspicion for verrucous carcinoma or other malignant entities. We also acknowledge that, in practice, a persistent verrucous lip lesion that fails to respond to conservative therapy may represent an invasive well-differentiated squamous cell carcinoma rather than VC, further underscoring the need for adequate sampling and careful histopathologic correlation with the clinical findings.

In such situations, an adequately deep or excisional biopsy is essential to sample the pushing invasion front and to avoid misclassification as a benign hyperkeratotic or papillomatous lesion. Once the diagnosis is established, wide local excision with histologically clear margins remains the mainstay of treatment. In our patient, this approach, combined with step-ladder advancement flap reconstruction, resulted in preservation of oral competence, symmetric lip contour, and the absence of recurrence during 18 months of follow-up.

Clear surgical margins and regular clinical surveillance for local recurrence and regional lymph node involvement are the key components of successful management of verrucous carcinoma of the lip. Increased awareness of the diagnostic limitations of superficial biopsies in persistent verrucous lip lesions may help clinicians avoid prolonged misdiagnosis and initiate timely surgical treatment, thereby minimizing morbidity and improving patient outcomes.

## Data Availability

The sharing of data is carried out in accordance with the consent provided by participants on the use of confidential data.

## Abbreviation

VC	verrucous carcinoma

## References

[1] Ackerman L.V. (1948). Verrucous carcinoma of the oral cavity. Surgery.

[2] Kristofelc N., Zidar N., Strojan P. (2023). Oral verrucous carcinoma: A diagnostic and therapeutic challenge. Radiol. Oncol..

[3] Pal U.S., Maurya H.K., Yadav S.K., Singh N., Mandhyan D., Mohammad S., Singh R.K. (2023). Protocol for treatment of oral verrucous carcinoma—A systematic review and meta-analysis. J. Oral Maxillofac. Pathol..

[4] Peng Q., Wang Y., Quan H., Li Y., Tang Z. (2016). Oral verrucous carcinoma: From multifactorial etiology to diverse treatment regimens (Review). Int. J. Oncol..

[5] Rekha K.P., Angadi P.V. (2010). Verrucous carcinoma of the oral cavity: A clinico-pathologic appraisal of 133 cases in Indians. Oral Maxillofac. Surg..

[6] Bouquot J.E. (1998). Oral verrucous carcinoma: Incidence in two US populations. Oral Surg. Oral Med. Oral Pathol. Oral Radiol. Endod..

[7] Kumar R.B.V., Raj A.C. (2012). Verrucous carcinoma of lip: An unusual presentation. Amrita J. Med..

[8] Chai K., Liu J., Xiao R., Zhang G., Zhan Y. (2023). A huge verrucous carcinoma of the lower lip reconstructed by double Abbe flap: A case report and literature review. Front. Oncol..

[9] Sun S.A., Lee K.E. (2014). Verrucous carcinoma of the lower lip: A case report. J. Oral Med. Pain.

[10] Leite A.F.S.D.A., Lanaro N.D., de Oliveira S.P., Santos-Silva A.R., Vargas P.A., Lopes M.A., Jorge J. (2018). Verrucous squamous cell carcinoma of the lower lip: A case report. Oral Surg. Oral Med. Oral Pathol. Oral Radiol..

[11] Nnko K.A., Newton R.C.C., Kahamba J.F., Mremi A., Tungu P.K. (2023). Management of huge verrucous carcinoma of lower lip: A case report at a tertiary hospital in northern Tanzania. Clin. Case Rep..

[12] Koch B.B., Trask D.K., Hoffman H.T., Karnell L.H., Robinson R.A., Zhen W., Menck H.R. (2001). National survey of head and neck verrucous carcinoma: Patterns of presentation, care, and outcome. Cancer.

[13] Wang N., Huang M., Lv H. (2020). Head and neck verrucous carcinoma: A population-based analysis of incidence, treatment, and prognosis. Medicine.

[14] Terada T. (2011). Verrucous carcinoma of the oral cavity: A histopathologic study of 10 Japanese cases. J. Maxillofac. Oral Surg..

[15] Kolokythas A., Rogers T.M., Miloro M. (2010). Hybrid verrucous squamous carcinoma of the oral cavity: Treatment considerations based on a critical review of the literature. J. Oral Maxillofac. Surg..

[16] de Carvalho Kimura T., Scatolim D.B., Henschel F.A.N., Veltrini V.C. (2022). Hybrid verrucous carcinoma: A wolf in sheep’s clothing. Case report and integrative review of 280 cases. Int. J. Odontostomatol..

[17] Srivastava N., Shetty A., Apparaju V., Goswami R.D. (2016). Surgical management of oral verrucous carcinoma and its recurrence: A case report and an update. Int. J. Med. Pharm. Case Rep..

[18] Ebrahimi A., Kalantar-Motamedi M.H., Ebrahimi A., Kazemi M., Shams A., Hashemzadeh H. (2016). Lip reconstruction after tumor ablation. World J. Plast. Surg..

[19] Moon B.M., Pae W.S. (2021). Reconstruction of a large lower lip defect using a combination of Abbe and staircase flaps: A case report. Arch. Craniofac. Surg..

